# Supplementary material for: Acceptability and usability of COVID-19 antigen self-test in populations under socioeconomic vulnerability in Brazil: A cross-sectional study
Source: PLOS Glob Public Health. 2026 Feb 5;6(2):e0005678. doi: 10.1371/journal.pgph.0005678 (PMC12875580; doi:10.1371/journal.pgph.0005678)
Supplement: Attachment — Submitted filename: Carta aos revisores.docx [file pgph.0005678.s003.docx]

**Reviewer #1**:

**Major comments**

**Introduction: In the title, the authors clearly highlight the fact that the study was conducted in “populations under socioeconomic vulnerability” as one of the study’s key contributions to the scientific literature. To enhance readability and to emphasize novel aspects of the analysis, I would suggest to shorten the introduction, removing especially any aspects that do not directly relate to the topic of “antigen-based self-testing for SARS-CoV-2 in vulnerable populations”. Particularly, I would suggest to merge the second and third paragraph, and to shorten this merged paragraph to a maximum of three sentences. I would also suggest to merge the fourth and fifth paragraph, again condensing this merged paragraph to a maximum of 3-4 sentences.**

Thank you for your thoughtful review. We have revised the introduction according to your comments and suggestions.

**Discussion: As for the introduction, the discussion should have a clear focus on interpreting the study’s results in the context of using COVID-19 self-testing in deprived populations. I would ask the authors to state how their results differ compared to (1) studies that were conducted in settings with high income and a high level of education, (2) studies that we done in similar settings as the present study. I am aware that the authors already touch upon these aspects, e.g., in line 356 to 360, but would suggest to make “COVID-19 self-testing in deprived populations” a stronger focus of the discussion, and further explore *why* results might differ to other / similar settings. Furthermore, I would appreciate it if the authors could discuss the above-mentioned aspects without further increasing the word count of the discussion. Lastly, as a minor note, the authors speak of “challenges” in the discussion on several occasions. I find the term “challenges” vague, and would instead suggest to specifically describe the most relevant challenges encountered.**

We revised the Discussion to address these points.

**Minor comments**

**Title: I would suggest to specify that the SARS-CoV-2 tests assessed in the study are *antigen* tests.**

We have revised the title according to your suggestion.

**Abstract: I would suggest to shorten the results section of the abstract along the lines of “… than in Rio de Janeiro (81.7%) (p<0.01). Participants showed difficulty interpreting test results, particularly the inconclusive with a positive mark (32.7% correct interpretations) and faint positive markers significantly reduced accuracy (below 40%). Accuracy in interpretation of test results was 89.6% overall, with moderate to substantial inter-rater agreement (Cohen’s kappa 0.56 overall, reaching 0.78 in some age groups). Over 90% of participants were willing to reuse and recommended the COVID-19 self-test. Higher age and lower educational status were associated with a reduced likelihood of obtaining a valid results and increased difficulties in test setup and interpretation.**

We have revised the Abstract according to your suggestion.

**Main** **text:** **Line 76: The word “a” seems out of place and should be removed.
- Line 135: Please replace “showed” by “shown”
- Table 3: please replace “ref =” by “reference: “ at all instances. Also, there seems to be a closing-bracket missing at the end of the
- Table 4: what is meant by “faixa etaria”? Please use English language instead
- Line 309: add “as” for “… such as education …”
- Line 355: I assume it should say “antigen” instead of “antibodies”?
- Line 385 to 387: would delete as it does not stem from the study’s results**

Thank you for your thoughtful review. We have revised the text according to accommodate the reviewer’s suggestions as much as possible.

**Conflict of interest: - I would ask the authors to be more specific in outlining their conflict of interest. In which way / to what extent was the work supported by WHO, UNITAID and the Brazilian Ministry of Health?**

The funders of the study had no role in study design, data collection, data analysis, data interpretation, or writing of the report. We updated the competing interests section to include this statement.

**Reviewer #2:**
**Major revisions**
**1. Please check grammar and spelling throughout the manuscript.**

An English native speaker carefully checked grammar and spelling throughout the manuscript.

**2. How representative do you think your study population is relative to the general population of the neighborhoods you are studying?**

Although our sample is not statistically representative, according to the 2019 Brazilian National Health Survey, approximately 76% of Brazilians rely exclusively on the public health system, with this proportion expected to be even higher in socioeconomically vulnerable areas. Therefore, we expect that our study population closely reflects the actual population living in the neighborhoods under study. We have addressed this point in the Discussion by adding a sentence.

**3. Where there any factors that could have possibly created the challenges highlighted by the study population in Rio de Janeiro compared to Salvador (ie, packaging, instructions, etc.)?**

Research teams received the same training in both cities. Participants also received the same information in both cities. Therefore, we could not find any factors such as packaging, instructions, that could explain these differences.

**4. Why is reporting cases an issue in Brazil? Please explain.**

We thank the reviewer for this comment. We have addressed this point in the Discussion by adding a sentence noting that the study did not assess the feasibility of reporting cases to epidemiologic surveillance authorities. This could be a challenge in Brazil, since data are reported by healthcare professionals rather than by individuals performing self-tests, indicating that a separate reporting channel would need to be established for self-tests.

**Minor revisions**
**1. I understand that the testing scenario was simulated, but is it accurate to assume that in real life participants who are likely sick and showing symptoms would be taking the test in the same environment that they are in the study? Would they be at a clinic with nurses there? Or would this likely happen at home alone? I would touch on this and how it might affect your results. This could also play into what you are saying about internet access to watch the YouTube video.**

We thank the reviewer for this comment. We agree that in real-life scenarios symptomatic individuals would likely perform self-tests at home rather than in a supervised or clinical setting. In Brazil, there is currently no formal policy for COVID-19 self-test distribution; however, if tests were provided through the public health system, they would likely be free and used at home, like other public health programs such as HIV testing. In our study, healthcare professionals acted only as observers and did not assist participants in performing or interpreting the tests. Therefore, we expect that the usability challenges observed would similarly occur in home-based settings, highlighting potential limitations in real-life implementation. We have addressed this point in the Discussion by adding a sentence.

**2. I think it is important to discuss where people access these tests and how that could play into usability and acceptability. Also, how are they accessible in terms of cost? Are people less likely to even bother getting a test due to their cost? Especially if they expect to not have a valid result?**

In Brazil, there is currently no formal policy for COVID-19 self-test distribution; however, if self-tests were provided through the public health system, they would likely be distributed free of charge and used at home, as has been done in other public health programs such as HIV testing. Therefore, we expect that cost would not be a barrier to access or affect usability and acceptability. We have added a sentence in the Discussion to clarify this point.

**3. Why do you expect your results differ from the study in Sao Paolo? Why is your study a necessary addition to the literature if there is already a study done in Brazil?**

We thank the reviewer for this comment. We revised the text to change “study conducted among 417 individuals in São Paulo” to “survey conducted among 417 individuals in São Paulo” to better reflect the methodological differences between the two studies.

Our study differs substantially from the survey conducted in São Paulo (https://pubmed.ncbi.nlm.nih.gov/36056299/). The São Paulo study used a population-based survey approach, in which respondents were approached at over 400 randomly selected street points to assess the acceptability of rapid SARS-CoV-2 antigen self-testing. In contrast, our cross-sectional study (January–May 2023) involved participants using a COVID-19 antigen self-test in a simulated real-world setting, guided by an instructional video, while being observed without interference from a healthcare professional. Our study assessed usability and the ability to correctly interpret results—outcomes not evaluated in the São Paulo survey—and acceptability was evaluated based on willingness to reuse, user experience, and recommendation to others. By evaluating the performance and interpretation of self-tests, our study provides complementary and necessary evidence for understanding real-world usability and acceptability in socioeconomically vulnerable populations.

**4. More discussion on the implications of these results is necessary. Can any of your results be generalized to future potential outbreak scenarios for other pathogens or maybe another pandemic? How can you ensure thoughtful implementation in your study group to minimize further disparities in this group? What would scale-up look like in a situation like this? Lay out what we need to do better.**

Thank you for the suggestion. This point has been addressed in the revised Conclusions, which now emphasize the risks of misinterpreting faint positives and outline concrete, equity-focused implementation steps (simplified multimodal instructions, assisted self-testing options, and monitoring of validity by age/education) to guide scale-up and future outbreaks.

**Reviewer #3:**

**General comments:**

**Review manuscript text for grammatical errors.**

An English native speaker carefully checked grammar and spelling throughout the manuscript.

**Abstract: The authors mention a simulated real-world setting in the abstract methods, the meaning of this was unclear until reading the main text. Participants were observed in the primary care setting by a healthcare provider. The authors could consider clarifying.**
In our study, participants were provided with COVID-19 self-tests and asked to use them independently, without any assistance from healthcare professionals. The professionals were present only as observers and did not intervene in performing or interpreting the tests. This setup mimics the real-world scenario in which individuals would typically use self-tests at home without professional support, but allows us to observe and systematically evaluate usability and accuracy in a controlled environment. Therefore, we refer to it as a “simulated real-world setting”: it reproduces the conditions of home use while enabling structured observation and data collection. We included further explanation in the “Procedures” part of the manuscript.

**In the abstract results, the mention of age and education several times as factors associated with misinterpretation of results feels repetitive. The last sentence of the results could be moved to the conclusion, for example.**

We have revised the Abstract according to your suggestion.

**Background:**
**The perspective of the background section feels like it is referencing a prior stage of the COVID-19 pandemic. The dynamics of the pandemic have largely shifted, and large-scale public health interventions are no longer in place. While the use of self-tests is still important and valuable as SARS-CoV-2 remains prevalent, the paper may be better framed as setting us up for future success with self-testing more broadly, particularly among populations of socioeconomic vulnerability. I would recommend adding some commentary on how this work adds to the future of self-testing strategies.**
We have revised the Background according to your suggestion.

**Methods:
It may be important to mention that the Panbio COVID-19 antigen self-test uses an** **anterior nasal swab, as nasopharyngeal swabs were commonly used in early stages of the pandemic.**

We thank the reviewer for this suggestion. We have added a sentence in the Methods section specifying that the Panbio COVID-19 antigen self-test uses an anterior nasal swab, in contrast to the nasopharyngeal swabs commonly used in the early stages of the pandemic.

**Methods are clearly described. Good use of logistic regression with VIF test of multicollinearity among the variables.**

We thank the reviewer for the positive feedback and appreciate the acknowledgment of our methodological approach.

**Results:
The results do not mention final variable selection from AIC in the logistic regression model. Do the variables shown in Table 3, with an aOR, correspond to the variables included in the multivariable model?**

Yes, the variables shown in Table 3 with adjusted odds ratios correspond to those included in the final multivariable logistic regression model after selection based on AIC. We have added a sentence in the Methods section to clarify this point.

**There was also no mention of the VIF results and multicollinearity between variables. Was any multicollinearity identified and were any variables removed from the regression model as a result?**

We thank the reviewer for this comment. No multicollinearity was identified among the variables, as assessed by the variance inflation factor (VIF), and therefore no variables were removed from the regression model. We revised the Results to report this information.

**Discussion:
The authors compared the study well with other literature. Conclusions drawn from the study are clear.
As was discussed, participants had difficulty interpreting self-test results with faint positive lines. Based on the instructional video linked to in the methods, they only show a self-test result with a strong positive line. As most participants relied on the video for instructions versus the leaflet, this may have been a factor in difficulty interpreting faint results. Does the leaflet show an example of a positive with a faint line? This could be important to mention in the discussion.**

Thank you for this helpful comment. We agree that this is an important point. Indeed, the instructional video did not clearly show all possible appearances of positive results, especially faint positive lines, which may have contributed to misinterpretation among participants. The leaflet, however, presented this information more clearly, including an example of a faint positive line. We have added a note in the discussion highlighting that future large-scale self-testing initiatives using video-based instructions should ensure that all possible result appearances are clearly illustrated to minimize user errors.

**Reviewer #4: This paper is well written and well-scoped to add some valuable data from Brazil on COVID self-testing**

**Far more information is needed about recruitment procedures and implementation:
• Why were the cities of Salvador and Rio de Janeiro selected?**

The cities of Salvador and Rio de Janeiro were selected because they were part of the broader project “Expansion of testing, quarantine, digital health, and telemonitoring strategies to tackle the COVID-19 pandemic in Brazil,” which included Primary Healthcare units located in neighborhoods with high socioeconomic vulnerability in both cities. We added this information to the article.

**• What primary healthcare facilities were chosen and why? How many did study staff recruit at?**

The study used a convenience sampling approach. Participants were purposively selected to ensure diversity in race (at least 50% Black and Pardo or mixed-race), gender (at least 50% women), and age (with a minimum of one-fifth representation in each group: 18–24, 25–34, 35–44, 45–59, and 60+ years). Primary Healthcare Centers (PHCs) were chosen because they served populations with these demographic characteristics and were in areas aligned with the project’s focus on communities under high socioeconomic vulnerability. We included this information in the text.

**• How were participants actually selected? From a line list? How were they contacted? Who did the contacting and what was their background? How were the variables of race, gender, and age assessed?**

Thank you for this helpful comment. We have added these details to the Methods section, clarifying how participants were approached at the PHC entrance by healthcare professionals, how eligibility and recruitment were conducted, and how race, gender, and age were self-reported during the initial contact.

• Convenience sampling and purposive sampling are two separate approaches, yet both are mentioned. This could use more explanation.

Thank you for this observation. The sampling approach combined elements of both convenience and purposive sampling. The Primary Healthcare Centers (PHCs) were selected by convenience, as they were part of the broader project and served populations with high socioeconomic vulnerability. Within these PHCs, participants were purposively recruited to ensure diversity in race, gender, and age, according to predefined quotas. We have clarified this distinction in the revised Methods section.

**• The paper is presented as being about “socioeconomic vulnerability”, but it is not clear why participants on the study fall in this category, or the locations of recruitment represent this category of people.**

Thank you for this valuable comment. We have added text and a reference in the Methods section to clarify why the selected areas and their residents are classified as being under high socioeconomic vulnerability.

**• Who were the staff conducting the surveys? What was their background?**

All study staff on site were healthcare professionals. We have revised the text to clarify that these professionals were also responsible for conducting the surveys.

**• Where did both recruitment and data collection happen? Was it the same time as the consent?**

Thank you for this comment. All procedures took place on the same day and at the same location. We have added this information to the Procedures section to make it clearer.

**• For participants who tested positive on the self-test, were procedures any different? Were they still asked the acceptability questions and was that process any different? I would expect these participants could have been going through emotional stress at this point**

Thank you for this important question. Yes, all participants, including those who tested positive on the self-test, answered the acceptability questions. In practice, the survey was conducted during the waiting period for the self-test result. Since participants were asymptomatic and had not sought testing services, the process generally did not generate significant emotional distress. Individuals with a positive result were referred to the Primary Healthcare Center’s standard of care for confirmatory testing and follow-up. We added this information to the text to make it clear.

**Statistical analysis:
• Why were the variables for adjustment chosen? Were others considered and rejected?**

We selected the socioeconomic variables available in the survey that were theoretically associated with the outcome based on existing literature. Other variables, such as religion, occupation type, and employment status, were not included because they are not reported as relevant predictors in the literature regarding this outcome.

**• No mention of sample size consideration is present. Was there any sample size goal at the start of the study? why did the study stop recruitment at 444 participants?**

We established an intentional sample of 440 participants, including 20% of loss.

Results
**• Table 1 is not all in English and the spacing of Table 2 makes it hard to read**

Thank you for your comment. Both tables have been carefully reviewed and corrected — Table 1 is now fully in English, and the formatting and spacing of Table 2 have been adjusted to improve readability.

**• The outcome of interest has 88% prevalence (obtained a valid self-test). I’m not convinced that logistic regression is an appropriate model choice given this high outcome prevalence, as the odds ratios may be overestimated. Why is this justified? I suggest authors look into instead using log binomial or Poisson regression for prevalence ratios to see if that’s more appropriate since the outcome is common**

Thank you for this valuable suggestion. Following your comment, we revised the analysis and replaced the logistic regression with a Poisson regression model with robust standard errors to estimate prevalence ratios. The manuscript has been updated accordingly to reflect this methodological change throughout the text and tables.

**• In table 5, I don’t think including the chi square test coefficient adds value**

Thank you for your observation. We agree that the chi-square test coefficient does not add interpretative value to the table. We therefore kept only the p-values, which are sufficient to indicate statistical significance between groups.

Discussion
**• More discussion can be added about why such differences were seen between the study locations, it would also help to include more information in the methods section about the differences in context between locations**
We included more information about the study locations in the methods section. Additionally, the differences in valid test results between Salvador and Rio de Janeiro in our study might be explained by the higher inclusion of men and individuals with more education in Salvador's participant group. We included this in the third paragraph of discussion.

**• More limitations to discuss are that these results are all in the context of supervision and further support from study staff – real world interpretation is limited without this help**

Thank you for your comment. Participants did not receive any assistance from the healthcare professionals during the self-test procedure. As described in the Methods section, the professionals were present only to observe and ensure participant safety, without providing any guidance or intervention. The only optional support available was that participants could read the instruction leaflet or watch the video tutorial again if they felt it was necessary. Healthcare professionals did not interact with participants while they performed the self-test.
