# Supplementary material for: Acceptability and usability of COVID-19 antigen self-test in populations under socioeconomic vulnerability in Brazil: A cross-sectional study
Source: PLOS Glob Public Health. 2026 Feb 5;6(2):e0005678. doi: 10.1371/journal.pgph.0005678 (PMC12875580; doi:10.1371/journal.pgph.0005678)
Supplement: Attachment — Submitted filename: Reply.docx [file pgph.0005678.s004.docx]

**Reply to reviewers:**

Reviewer #1: I very much appreciate the author’s thoughtful consideration of the reviewers' comments to the manuscript! It is clear that great effort has gone into the revision, and I don’t have any further comments.

As a side note, I noticed two linguistic aspects while reading the manuscript that the authors may wish to consider if they find them helpful:

- Line 107: add a full stop after ‘(PHC)’ and start the new sentence with ‘… services (PHC). One PHC …’

**Response: Corrected.**

- Line 127 / 128: what exactly is meant with individuals that ‘perchance tested positive’? Does this refer to individuals that were seeking COVID-19 related services and were initially excluded from the study, but were then included because of the positive result? Please clarify.

**Response: This refers to participants who tested positive for COVID-19 during the study. We excluded this sentence as this information is described in the methods section, lines 169-172.**

Reviewer #2: The authors have appropriately addressed all of my previous comments. Thank you.

**Response: Thanks.**

Reviewer #3: Authors have sufficiently addressed all comments.

As authors have changed their regression method from logistic to Poisson, this needs to be updated in the abstract.

**Response: We updated our abstract accordingly.**

Reviewer #4: Great adjustments to the analysis and content in the paper! Thank you for addressing comments.

**Response: Thanks.**
